# The impact of submaximal exercise during heat and/or hypoxia on the cardiovascular and monocyte HSP72 responses to subsequent (post 24 h) exercise in hypoxia

**DOI:** 10.1186/2046-7648-3-15

**Published:** 2014-09-29

**Authors:** Ben J Lee, Emma L Emery-Sinclair, Richard WA Mackenzie, Afthab Hussain, Lee Taylor, Rob S James, C Douglas Thake

**Affiliations:** 1 Sport and Exercise Science Applied Research Group, Coventry University, Coventry, UK; 2 Inflammation and Infection Group, School of Science and Technology, University of Westminster, London, UK; 3 Department of Sport and Exercise Sciences, University of Bedfordshire, Bedford, UK

**Keywords:** Cross-acclimation, Preconditioning, Humans, Cycling

## Abstract

**Background:**

The aims of this study were to describe the cellular stress response to prolonged endurance exercise in acute heat, hypoxia and the combination of heat and hypoxia and to determine whether prior acute exposure to these stressors improved cellular tolerance to a subsequent exercise bout in hypoxia 24 h later.

**Methods:**

Twelve males (age 22 ± 4 years, height 1.77 ± 0.05 m, mass 79 ± 12.9 kg, VO_2_ max 3.57 ± 0.7 L · min^-1^) completed four trials (30-min rest, 90-min cycling at 50% normoxic VO_2_ max) in normothermic normoxia (NORM; 18°C, F_I_O_2_ = 0.21), heat (HEAT; 40°C, 20% RH), hypoxia (HYP; F_I_O_2_ = 0.14) or a combination of heat and hypoxia (COM; 40°C, 20% RH, F_I_O_2_ = 0.14) separated by at least 7 days. Twenty-four hours after each trial, participants completed a hypoxic stress test (HST; 15-min rest, 60-min cycling at 50% normoxic VO_2_ max, F_I_O_2_ = 0.14). Monocyte heat shock protein 72 (mHSP72) was assessed immediately before and after each exercise bout.

**Results:**

mHSP72 increased post exercise in NORM (107% ± 5.5%, *p* > 0.05), HYP (126% ± 16%, *p* < 0.01), HEAT (153% ± 14%, *p* < 0.01) and COM (161% ± 32%, *p* < 0.01). mHSP72 had returned to near-resting values 24 h after NORM (97% ± 8.6%) but was elevated after HEAT (130% ± 19%), HYP (118% ± 17%) and COM (131% ± 19%) (*p* < 0.05). mHSP72 increased from baseline after HST_NORM_ (118% ± 12%, *p* < 0.05), but did not increase further in HST_HEAT_, HST_HYP_ and HST_COM_.

**Conclusions:**

The prior induction of mHSP72 as a result of COM, HEAT and HYP attenuated further mHSP72 induction after HST and was indicative of conferred cellular tolerance.

## Background

The acute physiological and biochemical responses to the environmental stressors of heat and hypoxia are well characterized when viewed in isolation [1-3], yet in reality stressors can be and are often experienced in combination. However, few studies have examined the physiological and biochemical effects of such stressors combined [4].

Acute heat and hypoxic exposures at rest and during exercise produce similar physiological, metabolic and cellular responses [1,3,5]. For example, heart rate and minute ventilation are elevated in comparison to the same absolute workload under temperate and normoxic conditions. Disturbances to redox balance, seen in response to both heat and hypoxia [6,7] and augmented by exercise, are potent stimuli for increases in heat shock protein concentrations, specifically heat shock protein 72 (HSP72), in humans [8]. This shared and transient response facilitates adaptation to chronic stress (acclimation) and potential cross-tolerance to subsequent diverse stressors [9,10]. In the relative short term, the heat shock response (HSR) can confer tolerance to future exposure to a stressor(s); this is termed preconditioning [11]. Preconditioning documented 1 h after stress insult has been termed “classical preconditioning” and that 1–2 days after stress insult, the “second window of protection” (SWOP) [10]. For example, prior exposure to a preconditioning heat stress is known to confer survival to an otherwise lethal heat shock in cell lines [12] and in both tissue-specific and whole-body models in rodents [13]. In humans, preconditioning may block pro-inflammatory cytokine pathways or alter cellular cytokine tolerance [14]. The HSR modulates cytokine signal transduction and gene expression by inhibiting translocation of nuclear factor-kappa B (NF-κB) to the nucleus, thus preventing the activation of the inflammatory cascade and increases in tumour necrosis factor alpha (TNF-α) and interleukin-6 (IL-6) (for review, see [14]). Furthermore, increased expression of heat shock factor 1 (HSF-1) increases the expression of anti-inflammatory interleukin-10 (IL-10) [15]. Human studies using acute exercise or heat acclimation protocols to increase HSP72 have failed to alter cytokine levels in *ex vivo* heat- or lipopolysaccharide (LPS)-treated cells [11].

Physiological strain drives the adaptive process [16]; thus, determining the magnitude of strain induced by defined levels of heat and hypoxia could potentially inform both training strategies and be used as an adjunct in maintaining and/or aiding the recovery of function from injury. For example, athletes recovering from injury may need to reduce mechanical loading but as a consequence reduce systemic physiological strain limiting the aerobic training stimulus [17]. The additional imposition of either heat or hypoxia would allow physiological strain to be maintained or increased during rehabilitation/recovery. Furthermore, the characterization of heat and hypoxic responses could also play a role in optimizing the management of movements of individuals or groups (e.g. military personnel) between different environmental settings. For example, individuals who are physiologically adapted to heat may tolerate moderately hypoxic environments better than non-acclimated individuals [18].

To date, no research has compared the physiological, HSP72, and cytokine responses to exercise performed at an absolute work intensity in both heat and hypoxia and the combination thereof. Neither has the impact of this prior exposure on subsequent tolerance to hypoxic exercise been investigated.

Therefore, the first aim of this study was to compare the magnitude of physiological and cellular HSP72 and pro/anti-inflammatory cytokine responses to individual and combined exposures to heat and hypoxia during prolonged moderate intensity exercise in young, moderately fit, non-cycle-trained adult males. It was hypothesized that the combination of heat and hypoxia would increase physiological and cellular strain when compared to the individual stressors alone and that greater physiological strain would produce an enhanced heat shock response. The second aim was to determine how the prior exposure to heat and hypoxia alone or in combination would impact upon the physiological and cellular responses to a subsequent hypoxic exposure, 24 h after this initial exercise bout. It was hypothesized that inducing the greatest levels of physiological strain and heat shock response after the initial exposure would enhance physiological and cellular tolerance to hypoxia 24 h later in the participant population studied.

## Methods

### Participants

Twelve healthy male participants (mean ± standard deviation: age 22 ± 4 years, height 1.77 ± 0.05 m, mass 79.0 ± 12.9 kg, estimated body fat 13.7% ± 4.3%, normoxic peak oxygen uptake (VO_2_ peak) 3.57 ± 0.70 L · min^-1^) volunteered and provided their informed consent to take part in this study, which was given ethical approval by Coventry University Ethics Committee. Participants attended the laboratory on nine separate occasions. The initial visit involved preliminary tests for resting haemoglobin (Hb) concentration and anthropometry to estimate body fat [19] followed by the assessment of lactate threshold and VO_2_ peak.

Peak oxygen consumption was determined using an incremental exercise test to volitional exhaustion on a cycle ergometer (Monark Ergomedic 874E, Monark Exercise AB, Vansbro, Sweden) whilst breathing room air. The test began at a workload of 70 W for 4 min and was then increased by 35 W every 4 min until a fingertip capillary blood lactate (Biosen C-Line Analyser, EKF Diagnostics, Barleben, Germany) value of >4 mmol · L^-1^ was reached. Thereafter, workload increased 35 W every 2 min until volitional exhaustion. A cadence of 70 rev · min^-1^ was maintained throughout. Expired gas was collected into 200-L Douglas bags during the last minute of every stage and subsequently analysed to determine CO_2_ and O_2_ content, using a Servomex infrared and paramagnetic gas analyzer (model 1400, Servomex, Crowthorne, UK), respectively, and gas volume, via a Harvard dry gas meter (Cranlea and Company, Birmingham, UK). VO_2_ peak was considered to be achieved if two of the following criteria were met: (i) a respiratory exchange ratio of >1.1, (ii) a heart rate greater than 95% of age predicted maximum (220 - age) and (iii) a final blood lactate value in excess of 8 mmol · L^-1^. This protocol has shown a CV of <1.5% for oxygen consumption in our laboratory.

### Experimental protocol

Participants were exposed to four experimental trials, normothermic normoxia (NORM; 20°C, 40% RH), heat (HEAT; 40°C, 20% RH), hypoxia (HYP; F_I_O_2_ ≈ 0.14, equivalent to ≈ 3,000 m, 20°C, 40% RH) and heat and hypoxia combined (COM; F_I_O_2_ ≈ 0.14, 40°C, 20% RH) using a randomized block design. An F_I_O_2_ of 0.14 (equivalent to ≈ 3,000 m above sea level) and a temperature of 40°C were chosen as they are reasonably close to acute habitable limits for non-acclimatized individuals and are often experienced in isolation on sojourns by athletic populations, adventure tourists and the military. Within each trial, participants sat for 30 min followed by 90 min of submaximal cycling exercise at 50% normoxic VO_2_ peak. Pilot work demonstrated that this absolute workload remained below lactate threshold in HEAT, HYP and COM for the 90-min duration [20]. Twenty-four hours after each trial, participants undertook a further 60 min of cycling at an intensity corresponding to 50% normoxic VO_2_ peak following 15 min seated rest under normothermic hypoxic conditions (F_I_O_2_ 0.14 ± 0.001). This was termed the hypoxic stress test (HST) and was conducted to determine whether prior acute exposure to each condition had conferred any detectable preconditioning effect (the HST trials 24 h after NORM, HEAT, HYP and COM are referred to as HST_NORM_, HST_HEAT_, HST_HYP_ and HST_COM_, respectively).

On each laboratory visit, participants provided a urine sample for the assessment of urine specific gravity (USG; visual refractometer, Index Instruments, Cambridge, Cambridgeshire, UK) and osmolality (Osmocheck, Vitech Scientific, Partridge Green, West Sussex, UK), weighed themselves nude to ±0.1 kg and inserted a rectal thermometer (Grant Instruments, Royston, UK) 10 cm past the anal sphincter. A heart rate monitor (Suunto t6c, Suunto, Vantaa, Finland) was fitted around the chest. Arterial Hb oxygen saturation (SpO_2_) was monitored throughout and recorded during respiratory gas collections using a finger-clip pulse oximeter (3100 WristOx, Nonin Medical, Inc., Plymouth, MN, USA). The sensor has a reported accuracy of ±2 digits (manufacturer’s guide). Whilst seated, skin thermistors (Grant Instruments) were attached, using micro-pore tape, to the upper arm, upper thigh, chest and calf to allow continuous monitoring of mean skin and body temperature [21].

During all trials and subsequent HSTs, participants breathed through a mouthpiece and 30-mm-diameter connector (Harvard Ltd, Edenbridge, UK) attached to a two-way non-rebreathable valve (Harvard Ltd, Edenbridge, UK). Ethylene clear vinyl tubing was used to connect the inspiratory side of the valve to a series of 1,000-L Douglas bags filled with hypoxic gas generated by an oxygen filtration device (Hypoxico HYP-123 hypoxicator, New York, NY, USA). During normoxic trials, the valve was left open to the ambient air. Expired gas was collected into 200-L Douglas bags for 60 s every 10 min. After each expired gas collection, participants reported overall rating of perceived exertion (RPE) and thermal sensation (TS). Cardiac output (CO), stroke volume (SV) and ${a‒\bar{v}O}_{2}$ difference were estimated according to the equation of Stringer et al. [22]. The physiological strain index (PSI) was calculated using heart rate and rectal temperature and is reported on a scale of 0 (no strain) to 10 (very high strain) as described by Moran et al. [23].

**Figure 1:**
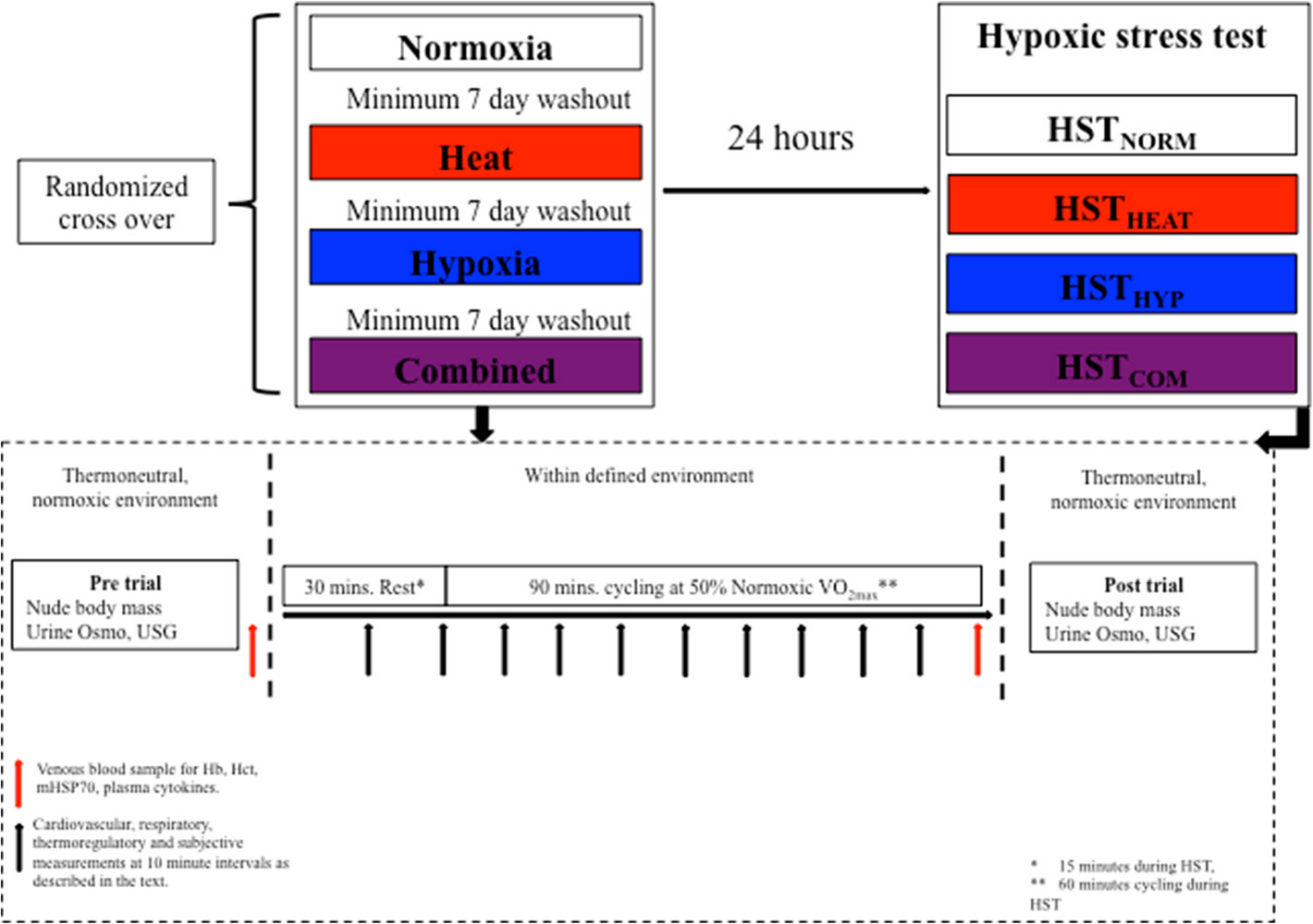
**Experimental schematic.** Experimental design and data collection timings during the initial environmental exposure and the subsequent hypoxic stress test 24 h later.

Resting venous blood samples were collected from an antecubital vein into potassium EDTA vacutainers (VACUETTE^®^, Greiner Bio-One, Stonehouse, UK) for the assessment of monocyte heat shock protein 72 (mHSP72), TNF-α, IL-6 and IL-10 following a 15-min seated rest period in normothermic normoxia. Post exercise, samples were collected immediately upon cessation of exercise with participants still seated on the ergometer and exposed to the specific conditions of the trial. Measurements of Hb and haematocrit were made to determine plasma volume according to the methods of Dill and Costill [24]. Details of the experimental method and timings of measurements throughout this investigation can be seen in Figure 1.

### Inflammatory/anti-inflammatory cytokines

Plasma TNF-α, IL-10 and IL-6 were determined independently using enzyme-linked immunosorbent assays (ELISA MAX, BioLegend, London, UK) with a sensitivity of 2, 2 and 4 pg · mL^-1^, respectively. Data were corrected for any changes in plasma volume.

### Flow cytometry analysis of monocyte heat stress protein 72

Measurement of mHSP72 has been detailed elsewhere [7,10,25]. Briefly, cells obtained after red cell lysis were fixed and permeabilized (AbD Serotec, Oxford, UK), and an isotype-matched negative control (FITC, AbD Serotec) or anti-HSP72 antibody (SPA-810, Assay Designs, Enzo Life Sciences, Inc., Farmingdale, NY, USA) was added to the same final concentration and then incubated for 30 min in the dark. Samples were then analysed by flow cytometry (BD FACSCalibur, BD Biosciences, San Jose, CA, USA) with monocytes gated by forward/side scatter properties and further discriminated by CD14 expression. Mean fluorescence intensity (MFI) was then calculated using CellQuest software (BD Biosciences) with a total of 15,000 cells counted. Results are presented as the ratio of MFI gained with the anti-HSP72 antibody to that obtained with the isotype-matched negative control and as percentage change from the resting value obtained at the beginning of each trial [26].

### Statistical analysis

All statistical analyses were performed using SPSS, version 20 (IBM, Armonk, NY, USA). Data were checked for normal distribution prior to analysis. Sphericity was checked with Mauchly’s sphericity test, and when necessary, the Huynh-Feltd method was applied to the *F*-ratio to correct for sphericity violations. All data are presented as mean ± SD for *n* = 12, with statistical significance set at *P* < 0.05.

### Initial environmental exposure and hypoxic stress test

Two-way repeated measures ANOVAs (condition by time) were performed to determine differences between environmental conditions both throughout rest and during exercise. Resting data were analysed separately from exercise data. Exercise data were comprised of measurements made at 10, 20, 30 and 40 min, and the final value was recorded upon cessation of exercise for each participant (five time points) for the initial environmental exposure and the HST. Data were further explored for the HST trial by comparing the percentage change in physiological data collected during HST_NORM_ with all other experimental HSTs via two-way repeated measures ANOVAs (condition by time). Alterations in mHSP72, plasma TNF-α, plasma IL-6, and plasma IL-10 were analysed via a two-way repeated measures ANOVA. mHSP72 was analysed as a percentage change from each trial’s initial baseline value obtained on day 1 [10]. All main effects were explored using Tukey’s HSD test. Effect sizes were calculated for mean exercising HST data using Cohen’s *D*, with the NORM condition acting as the control condition.

## Results

### Hydration state

All participants were euhydrated prior to the start of each experimental trial, with USG < 1.020 and *U*_osmo_ < 300 mOsmol/kg. Nude body mass did not vary prior to any experimental condition on day 1 (NORM 79.2 ± 13.8 kg, HEAT 79.2 ± 12.8 kg, HYP 79.3 ± 13.8 kg, COM 79.3 ± 13.1 kg).

### Cardiorespiratory responses at rest

The only physiological variables altered by the resting environmental exposures were heart rate, SpO_2_, respiratory exchange ratio (RER), *T*_skin_ and *T*_body_. All resting cardiovascular, respiratory and thermoregulatory data is presented in Tables 1 and 2.

**Table 1:** Resting cardiovascular and metabolic responses to acute physiological stressors

	**Baseline**	**15 min**	**30 min**		**15 min**	**30 min**
Heart rate (beats · min^-1^)				VO_2_ (L · min^-1^ STPD)		
Normoxia	69 ± 10	65 ± 10	64 ± 10	Normoxia	0.32 ± 0.08	0.30 ± 0.07
Heat	71 ± 14	73 ± 12*	75 ± 13*	Heat	0.31 ± 0.07	0.32 ± 0.10
Hypoxia	63 ± 9	69 ± 7	74 ± 8	Hypoxia	0.33 ± 0.06	0.35 ± 0.07
Combined	68 ± 9	80 ± 12*	82 ± 10*	Combined	0.34 ± 0.07	0.33 ± 0.08
SpO_2_ (%)				Cardiac output (L · min^-1^ STPD)		
Normoxia	98 ± 1	97 ± 1	97 ± 2	Normoxia	5.6 ± 1.3	5.4 ± 0.8
Heat	98 ± 1	97 ± 1	97 ± 1	Heat	5.6 ± 1.0	5.9 ± 1.6
Hypoxia	98 ± 1	90 ± 2*	89 ± 3*	Hypoxia	5.4 ± 1.1	5.7 ± 1.0
Combined	97 ± 1	91 ± 2*	89 ± 3*	Combined	5.5 ± 1.3	5.7 ± 1.6
*V*_E_ (L · min^-1^ BTPS)				Stroke volume (mL · beat^-1^)		
Normoxia	-	13.8 ± 4.4	13.3 ± 2.8	Normoxia	87 ± 18	86 ± 13
Heat	-	13.8 ± 3.7	14.4 ± 4.7	Heat	78 ± 12	79 ± 24
Hypoxia	-	15.0 ± 4.3*	14.5 ± 3.6	Hypoxia	80 ± 18	78 ± 19
Combined	-	13.7 ± 2.9	13.1 ± 2.8	Combined	70 ± 15	68 ± 16
*V*_E_ (L · min^-1^ STPD)				${a‒\bar{v}O}_{2}$ difference (mL · L^-1^)		
Normoxia	-	11.3 ± 3.6	10.8 ± 2.2	Normoxia	6.8 ± 0.5	6.8 ± 0.2
Heat	-	11.3 ± 3.0	11.7 ± 3.8	Heat	6.9 ± 0.2	6.9 ± 0.3
Hypoxia	-	12.7 ± 4.8	12.7 ± 3.9	Hypoxia	6.8 ± 0.3	6.9 ± 0.4
Combined	-	11.5 ± 2.7	11.0 ± 2.7	Combined	6.8 ± 0.2	6.9 ± 0.3
VO_2_ (L · min^-1^ STPD)				RER		
Normoxia	-	0.39 ± 0.11	0.37 ± 0.06	Normoxia	0.83 ± 0.07	0.82 ± 0.15
Heat	-	0.39 ± 0.07	0.41 ± 0.12	Heat	0.79 ± 0.10	0.79 ± 0.10
Hypoxia	-	0.37 ± 0.08	0.39 ± 0.08	Hypoxia	0.90 ± 0.09*	0.88 ± 0.07*
Combined	-	0.38 ± 0.10	0.39 ± 0.13	Combined	0.89 ± 0.08	0.85 ± 0.09

*p < 0.05 compared to the corresponding time point in the normoxic condition.

**Table 2:** Resting thermoregulatory measurements

	**Baseline**	**15 min**	**30 min**
Core temperature (°C)			
Normoxia	37.2 ± 0.3	37.2 ± 0.2	37.2 ± 0.2
Heat	37.2 ± 0.3	37.2 ± 0.4	37.3 ± 0.3
Hypoxia	37.3 ± 0.3	37.2 ± 0.2	37.1 ± 0.2
Combined	37.3 ± 0.3	37.3 ± 0.3	37.3 ± 0.3
Mean skin temperature (**°**C)			
Normoxia	31.1 ± 0.6	31.2 ± 0.9	31.2 ± 0.8
Heat	32.2 ± 0.7	34.9 ± 0.5*	34.9 ± 0.5*
Hypoxia	31.0 ± 1.0	31.2 ± 0.9	31.1 ± 1.0
Mean body temperature (**°**C)	31.6 ± 0.7	34.3 ± 1.1*	34.5 ± 1.1*
Normoxia	35.9 ± 0.2	35.9 ± 0.2	35.9 ± 0.2
Heat	36.1 ± 0.3	36.7 ± 0.3*	36.8 ± 0.2*
Hypoxia	36.1 ± 0.2	36.1 ± 0.2	35.9 ± 0.3
Combined	36.1 ± 0.2	36.7 ± 0.3*	36.7 ± 0.3*

*p < 0.05 compared to NORM and HYP at the corresponding time point.

### Exercise performance

**Figure 2:**
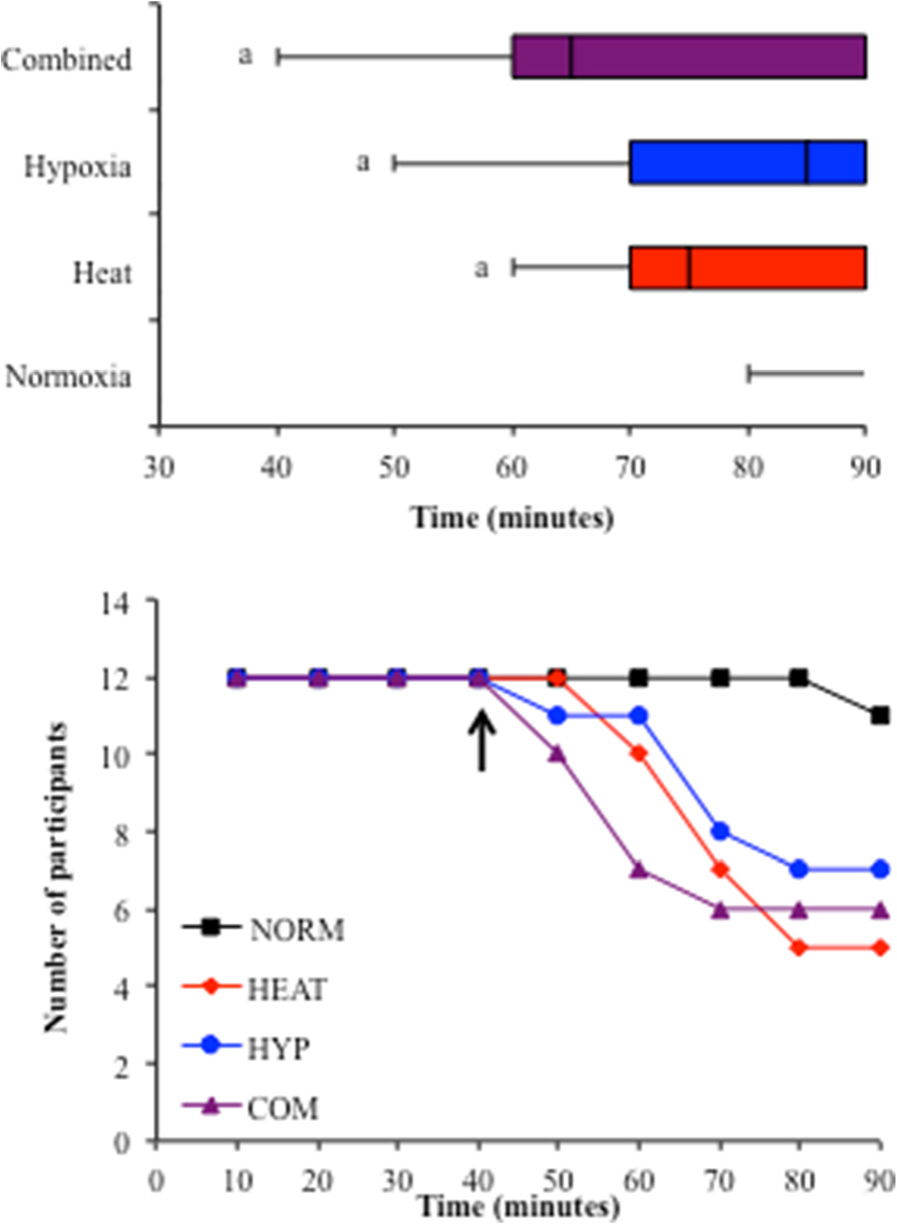
**Exercise times to exhaustion during each condition.** The *top panel* displays exercise times until exhaustion during the four experimental trials. Data show the first, second (median) and third interquartile ranges (*coloured boxes*) and shortest achieved time in each condition. *a* = significantly different from NORM (*p <* 0.01). The *bottom panel* displays the participant dropout during each condition. The *arrow* denotes the point at which pairwise comparisons were made for statistical analysis between the 12 participants.

Participants completed all trials at a workload of 146 ± 19 W. Of the 12 participants, 2 completed the 90-min exercise bout in all trials and 4 participants failed to complete the 90-min bout in any of the environmental conditions. Time to exhaustion was significantly reduced in COM (73 ± 19 min, *p =* 0.01), HYP (81 ± 13 min, *p* = 0.04) and HEAT (78 ± 12 min, *p* = 0.005) compared to NORM where all but one subject completed the full 90 min of exercise (89 ± 3 min) (Figure 2).

Times to cessation of exercise for the four participants that were unable to complete any of the environmental stressor trials were HEAT 70 ± 8.1, HYP 67.5 ± 12.6 and COM 52.5 ± 9.6 min. These four participants had a relatively low level of aerobic fitness (35–40 mL · kg · min^-1^). A trend was observed between total time completed during all four trials and relative aerobic capacity for all 12 participants (*r* = 0.55, *p =* 0.06). A further three participants failed to complete HEAT despite finishing the COM trial (60, 70 and 82 min), and one participant failed to complete HYP (72 min) despite completing COM. When participants were separated into trained (>50 mL · kg · min^-1^, 55.8 ± 5.5 mL · kg · min^-1^; *n* = 6) and untrained (<40 mL · kg · min^-1^, 38 ± 2.4 mL · kg · min^-1^; *n* = 6), the effects of aerobic fitness become more apparent. The trained group completed 90 ± 0, 80 ± 12, 90 ± 0 and 87 ± 8 min of exercise in NORM, HEAT, HYP and COM, respectively, whereas the untrained group completed 88 ± 4, 77 ± 12, 72 ± 13 and 60 ± 16 min of exercise in NORM, HEAT, HYP and COM, respectively. Pearson correlations, adjusted for multiple comparisons, revealed that maximal aerobic capacity was positively related to performance time in the HYP (*r =* 0.699, *p* = 0.01) and COM (*r =* 0.598, *p* = 0.04) conditions, but no such relationship existed for HEAT (*r* = -0.027, *p* = 0.933). Table 2 shows all physiological values upon termination of exercise in each condition. At the end of exercise, the percentage of normoxic VO_2_ peak was 57% ± 14%, 60% ± 9%, 59% ± 15% and 57% ± 11% in NORM, HEAT, HYP and COM, respectively.

### Cardiorespiratory responses to exercise

**Figure 3:**
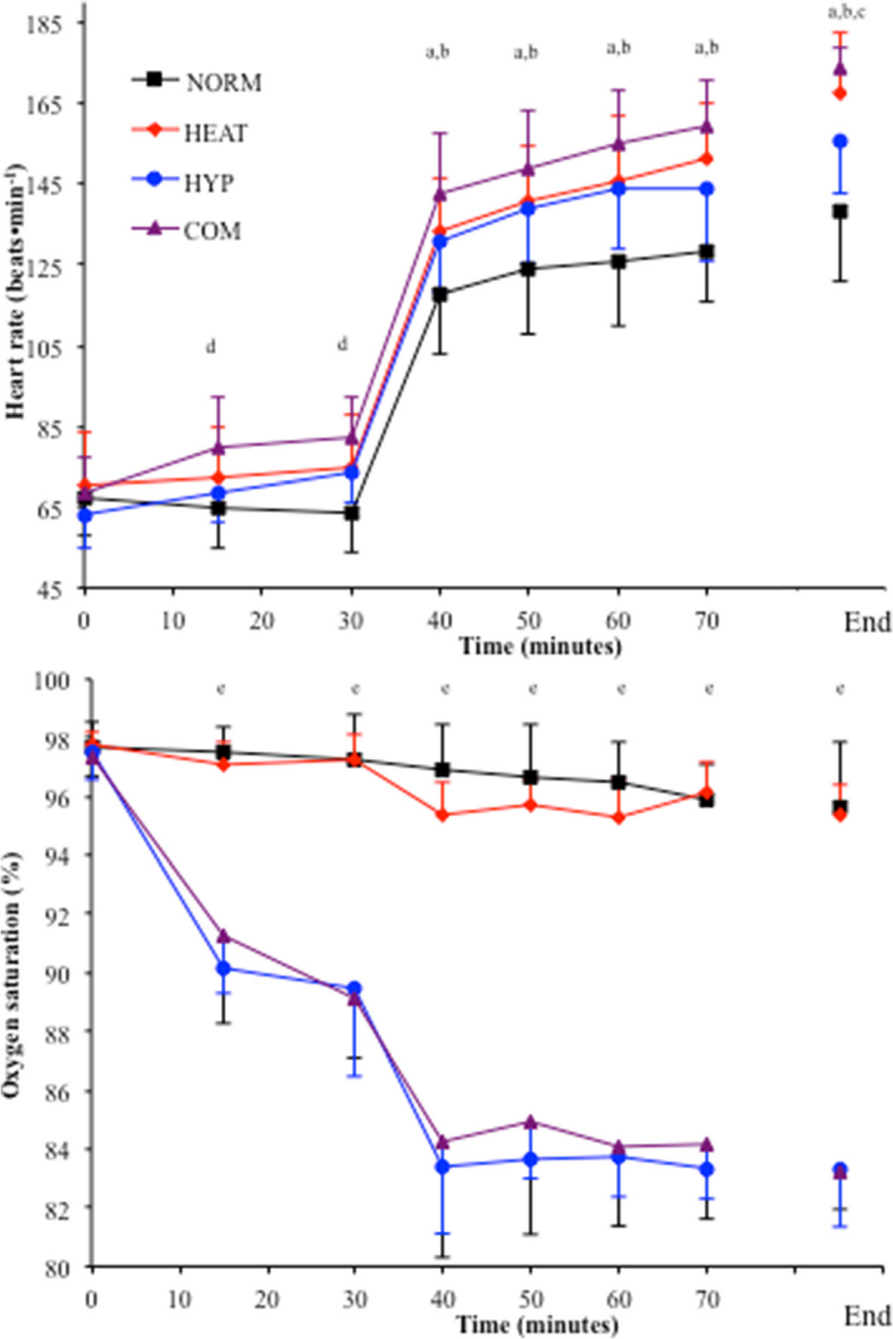
**Heart rate and SpO**_**2 **_**responses to acute rest and exercise under heat or hypoxic stress.** The *top panel* shows the heart rate response during each trial; the *bottom panel* shows the SpO_2_ response during each trial. *a* = HEAT, HYP and COM different from to NORM (*p* < 0.05), *b* = COM different from HYP (*p* < 0.05), *c* = HEAT different from HYP (*p* < 0.05), *d* = COM different from NORM (*p* < 0.05), *e* = HYP and COM different from NORM and HEAT (*p* < 0.01).

Heart rate varied between conditions throughout exercise and was lowest in NORM (*p* < 0.01 vs. HEAT, HYP and COM) and tended to be greatest in COM (*p* < 0.05 vs. HYP). HR did not vary between HEAT and HYP until termination of exercise, where HR was higher in HEAT (*p* < 0.05) and COM (*p* < 0.01) compared with HYP (Figure 3). During exercise, SpO_2_ was lower at each time point in HYP and COM compared to NORM and HEAT (*p* < 0.01). Upon termination of exercise, SpO_2_ was lower in HYP and COM compared to NORM and HEAT (*p* < 0.01; Table 3). No difference between HYP and COM was found at any time point (Figure 3).

**Table 3:** Cardiovascular, metabolic, thermoregulatory and subjective data upon termination of exercise across experimental conditions

	**Normoxia**	**Heat**	**Hypoxia**	**Combined**
Cardiovascular				
HR (beats · min^-1^)	138 ± 17	168 ± 15*^,^**	156 ± 13*	174 ± 5*^,^**
SpO_2_ (%)	96 ± 2	95 ± 1	83 ± 1*^,^***	83 ± 1*^,^***
Cardiac output (L · min^-1^)	16.9 ± 2.5	17.6 ± 2.8	17.3 ± 2.5	17.4 ± 3.2
Stroke volume (mL · beat^-1^)	126 ± 33	106 ± 18	112 ± 23	101 ± 16
${a‒\bar{v}O}_{2}$ difference	10.95 ± 2	11.96 ± 1	11.97 ± 1.5	11.7 ± 1.2
Plasma volume change (%)	-2.0 ± 5.8	-3.2 ± 10	-1.7 ± 7.3	-2.4 ± 5.4
Metabolic				
VO_2_ (L · min^-1^)	1.98 ± 0.37	2.09 ± 0.33	2.06 ± 0.42	2.02 ± 0.49
VCO_2_ (L · min^-1^)	1.71 ± 0.34	1.80 ± 0.29	1.91 ± 0.36	1.83 ± 0.35
RER	0.87 ± 0.09	0.86 ± 0.06	0.93 ± 0.07*^,^***	0.92 ± 0.09*^,^***
*V*_E_ STPD (L · min^-1^)	40.5 ± 5.90	46.4 ± 9.30*	53.0 ± 12.2*	52.2 ± 10.70*
*V*_E_ BTPS (L · min^-1^)	49.6 ± 7.30	56.9 ± 11.8*	63.3 ± 14.1*	62.1 ± 9.90*
Thermoregulatory				
*T*_core_ (**°**C)	37.8 ± 0.3	38.7 ± 0.5*^,^**	38.0 ± 0.3	38.6 ± 0.4*^,^**
*T*_skin_ (**°**C)	31.4 ± 2.0	36.2 ± 0.9*^,^**	32.1 ± 1.3	35.8 ± 1.0*^,^**
*T*_body_ (**°**C)	36.5 ± 0.6	38.1 ± 0.4*^,^**	36.9 ± 0.3	38.1 ± 0.4*^,^**
Sweat rate (L · min^-1^)	0.48 ± 0.2	1.05 ± 0.2*^,^**	0.46 ± 0.2	0.91 ± 0.2*^,^**
PSI (AU)	4.5 ± 0.9	7.6 ± 1.5*^,^**	5.2 ± 0.7	7.6 ± 1.1*^,^**
Perceptual				
RPE (AU)	14 ± 2	17 ± 2*	17 ± 2*	17 ± 2*
TS (AU)	5 ± 1	7 ± 1	6 ± 1	7 ± 1

*Significantly different from NORM; **significantly different from HYP; ***significantly different from HEAT (*p <* 0.05).

No main effect for condition was found for oxygen consumption (*p* = 0.88) or carbon dioxide production (*p* = 0.21). RER was higher in HYP compared to NORM (*p* < 0.01) and HEAT (*p* < 0.01). RER was higher at the end of exercise in HYP and COM compared to NORM and HEAT (*p* < 0.05). *V*_E_ BTPS was higher in HYP and COM compared to NORM (*p* < 0.001). There was a trend for *V*_E_ BTPS to be higher in COM compared to HEAT (*p* = 0.06). *V*_E_ BTPS was higher at the end of exercise in HYP and COM compared to NORM (*p* < 0.01), but not significantly higher compared to HEAT (*p* > 0.05) (Table 3).

### Thermoregulatory responses and physiological strain index

**Figure 4:**
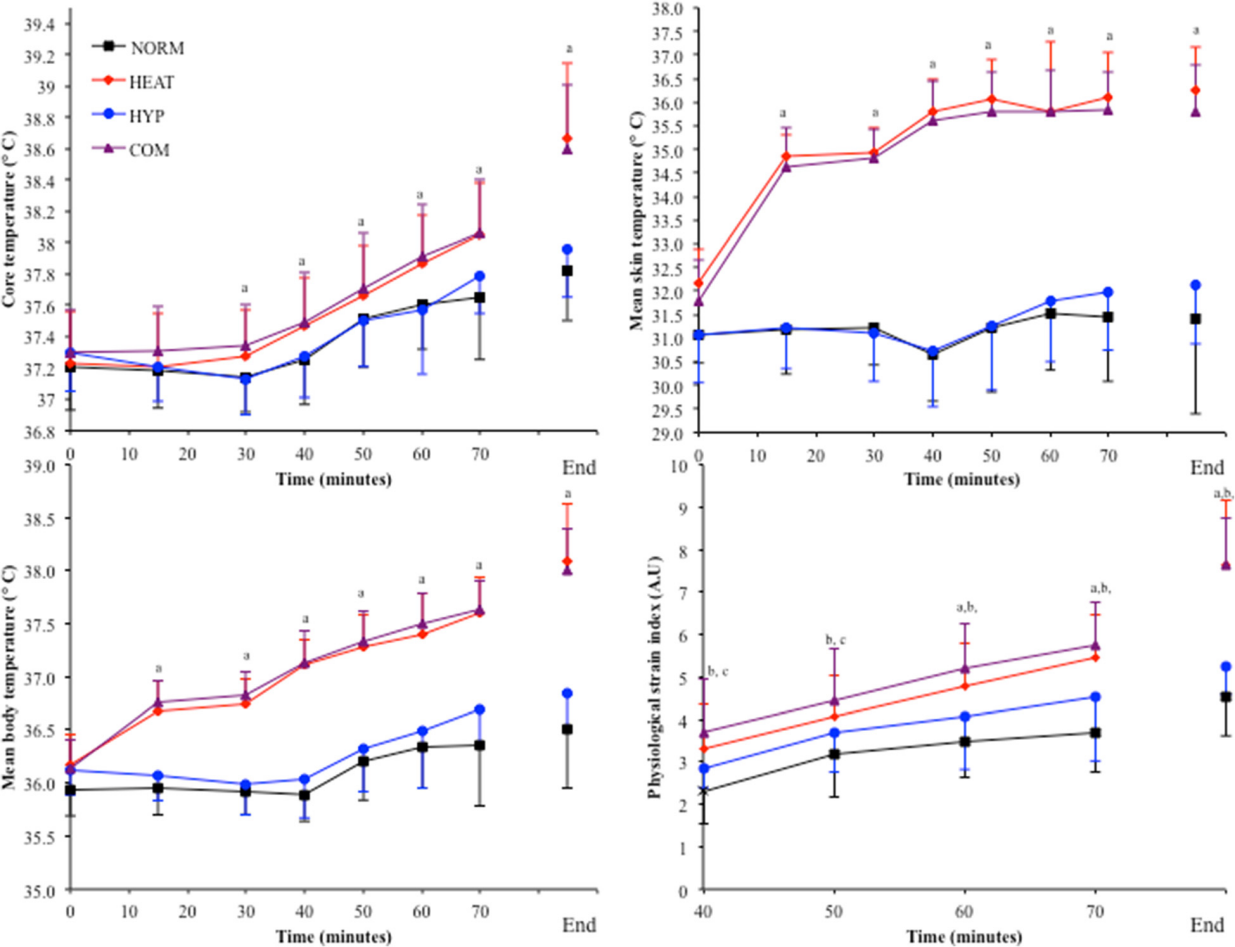
**Thermoregulatory responses to each experimental trial.** The *top left panel* shows core temperature during each trial, the *top right panel* shows mean skin temperature during each trial, the *bottom left panel* shows mean body temperature during each trial and the *bottom right panel* shows physiological strain index during each trial. *a* = HEAT and COM different from NORM and HYP (*p* < 0.01), *b* = HEAT, HYP and COM different from NORM (*p* < 0.05), *c* = COM different from HYP (*p* < 0.05).

*T*_core_ increased throughout exercise and was elevated upon exercise termination in all trials (*p* < 0.001). *T*_core_ was greater at each time point in HEAT and COM compared to NORM and HYP (*p* < 0.001; Figure 4). During NORM and HYP, *T*_core_ rose during the initial 20 min of exercise before reaching a plateau at 30 min. A similar response was observed for mean skin temperature, which was higher at each time point, and upon exercise termination in HEAT and COM compared to NORM and HYP (*p* = 0.006; Figure 4).

Mean body temperature was higher at each time point throughout exercise and upon the cessation of exercise in HEAT and COM compared to NORM and HYP (*p* < 0.001). Physiological strain was higher throughout exercise in HEAT, HYP and COM compared to NORM (*p* < 0.05) and all higher than NORM upon exercise termination (*p* < 0.01). Compared to HYP, PSI was higher throughout exercise in the COM trial (*p* < 0.01) and higher during the HEAT trial from 30 min through to exercise termination (*p <* 0.01) (Figure 4). Sweat rates and percent change in body mass were higher during HEAT and COM compared to NORM and HYP (*p* < 0.001). Plasma volume did not vary at rest (*p* = 0.169) or post exercise (*p* = 0.147) between trials (Table 3).

### Ratings of perceived exertion and thermal comfort

RPE increased in a linear fashion throughout all of the trials and was higher throughout exercise in COM compared to NORM and HYP at 10 and 20 min (*p* < 0.05). RPE was significantly higher at the end of exercise in all experimental conditions compared to NORM (*p* < 0.01, Table 3); however, no difference was found between the other environmental stressors upon exercise termination (*p* > 0.05). Thermal sensation was higher at all time points in HEAT, HYP and COM compared to NORM (*p* < 0.01; Table 3). Upon exercise termination, thermal sensation was significantly higher in HEAT (*p* < 0.01), HYP (*p* < 0.05) and COM (*p* < 0.01) compared to NORM and higher in HEAT and COM compared to HYP (*p* < 0.01).

### Monocyte HSP72 responses to acute environmental exposure

There was no difference in resting mHSP72 prior to exposure to any experimental conditions (*p* > 0.05). mHSP72 increased post exercise in HYP (126% ± 16%), HEAT (153% ± 14%) and COM (161% ± 32%) (*p* < 0.001), but not NORM (107% ± 5.5%, *p* > 0.05). Post exercise values were higher following HEAT and COM compared to HYP (*p* < 0.01). Post exercise mHSP72 did not vary between HEAT and COM. Post exercise mHSP72 was not related to final core temperature in NORM (*r =* -0.214, *p* = 0.505) and HEAT (*r* = 0.199, *p* = 0.536), whereas a relationship between final core temperature and mHSP72 was observed in HYP (*r* = 0.562, *p* = 0.057) and COM (*r* = 0.539, *p* = 0.071).

**Figure 5:**
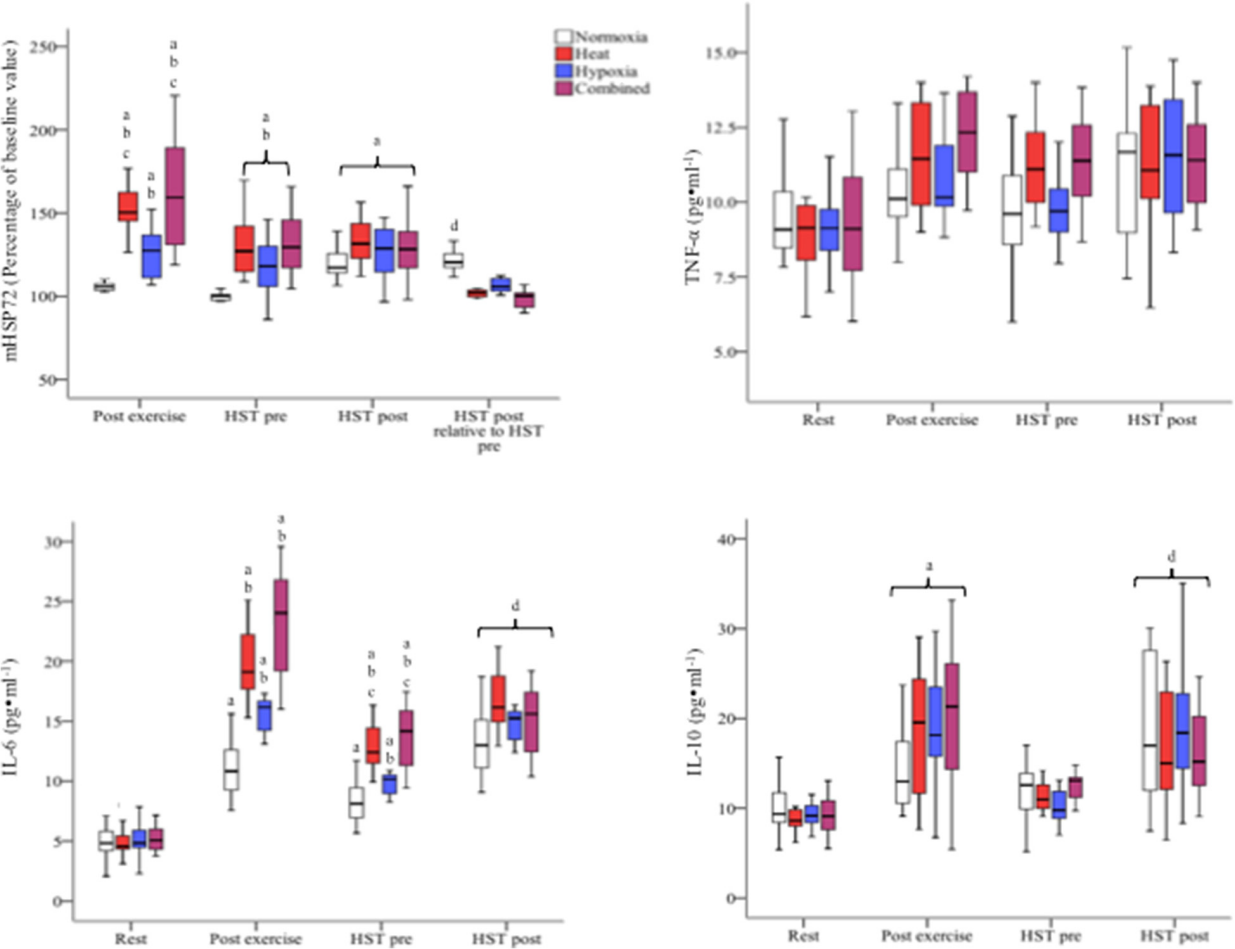
**Monocyte HSP72 and cytokine responses to each experimental trial.** The *top left panel* shows mHSP72 expression over time for each of the four experimental conditions. Data at the end of exercise (day 1), pre-HST and post-HST are expressed as a percentage of the control value [26]. The *top right panel* shows TNF-α before and after each experimental trial. The *bottom left panel* shows IL-6 and the *bottom right panel* shows IL-10 before and after each experimental trial. Data show the first, second (median) and third interquartile ranges (*coloured boxes*) and the lowest and highest values in each condition (*T bars*). *Letters* represent significant differences between means (*p* < 0.05). *Error bars* represent the standard error of the mean. *a* = different from baseline (*p* < 0.05), *b* = different from NORM (*p* < 0.05), *c* = different from HYP (*p* < 0.05), *d* = different from pre-HST to post-HST.

### Plasma pro/anti-inflammatory cytokines

Resting TNF-α, IL-10 and IL-6 did not vary between conditions (*p* > 0.05; Figure 5). Plasma TNF-α was elevated after exercise (*p* = 0.025) and did not vary between conditions (*p* = 0.43). Plasma IL-6 was increased as a result of exercise in all trials (*p* < 0.01). HEAT, HYP and COM each produced greater elevations in IL-6 compared to NORM, with post exercise concentrations in IL-6 higher in HEAT and COM compared to HYP (*p <* 0.001). Plasma IL-10 increased post exercise in all conditions, with the magnitude of increase being greater following exercise in HEAT, HYP and COM compared to NORM (*p* < 0.001).

### Post-24-h HST responses

Exercise times for the HST were not different between trials. Only one participant was unable to complete the full 60-min exercise in each trial. Participant 6 completed 46, 48 and 46 min of exercise in HST_NORM_, HST_HEAT_ and HST_HYP_, respectively. Results were therefore analysed using pairwise comparisons up to 40 min, with the final values obtained at the end of each test also included in the analysis.

### Cardiorespiratory responses to the HST

**Figure 6:**
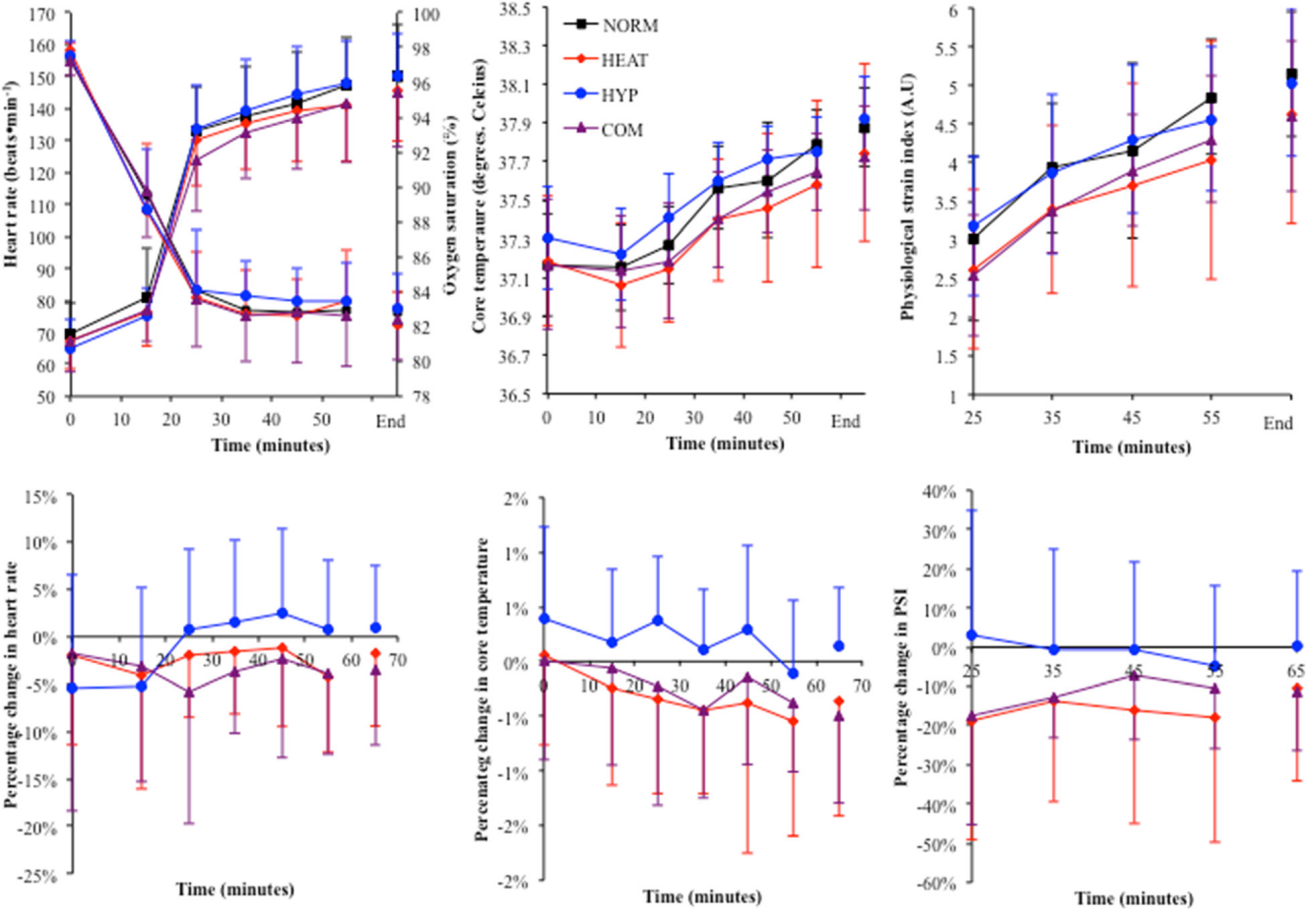
**Cardiovascular and thermoregulatory responses to each HST.** The *top left panel* shows the heart rate and SpO_2_ responses to each HST. The *top middle panel* shows the core temperature response to each HST. The *top right panel* shows the physiological strain index during each HST. The *bottom panels* represent the percentage difference in heart rate, core temperature and PSI, respectively, in relation to the HST_NORM_ trial.

The previous days exposure had no effect on any resting variable (*p* > 0.05). Exercising HR had a tendency to be lower in HST_COM_ and HST_HEAT_ compared to HST_NORM_. HR was ≈ 4 and 5 beats · min^-1^ lower at the end of exercise in HST_HEAT_ and HST_COM_ compared to HST_NORM_ and HST_HYP_, respectively (*p* = 0.08; Figure 6). Small effect sizes were observed for both HST_HEAT_ (*d =* -0.23) and HST_COM_ (*d =* -0.41), but not HYP (*d* = 0.09). SpO_2_ during rest, exercise and at the end of exercise was also similar between trials (*p* > 0.05; Figure 6). *V*_E_ (BTPS and STPD), VO_2_, VCO_2_ and RER did not vary between trials during exercise or at completion of the HST (*p* > 0.05). Table 4 shows the end point data for each HST.

**Table 4:** Cardiovascular, metabolic, thermoregulatory and subjective data upon termination of each hypoxic stress test

	**HST**_**NORM**_	**HST**_**HEAT**_	**HST**_**HYP**_	**HST**_**COM**_
Cardiovascular				
HR (beats · min^-1^)	150 ± 16	146 ± 16	150 ± 13	145 ± 17
SpO_2_ (%)	83 ± 2	82 ± 2	83 ± 2	82 ± 2
Cardiac output (L · min^-1^)	16.9 ± 2.7	15.9 ± 2.6	16.1 ± 2.6	16.0 ± 2.8
Stroke volume (mL · beat^-1^)	117 ± 31	110 ± 22	111 ± 27	113 ± 26
${a‒\bar{v}O}_{2}$ difference (mL · L^-1^)	11.1 ± 0.9	11.1 ± 1.4	11.1 ± 1.2	11.0 ± 0.9
Plasma volume (% change)	1.8 ± 7.6	8.3 ± 7.7	2.1 ± 4.7	3.1 ± 6.4
Metabolic				
VO_2_ (L · min^-1^)	1.89 ± 0.41	1.77 ± 0.41	1.79 ± 0.32	1.75 ± 0.42
VCO_2_ (L · min^-1^)	1.85 ± 0.33	1.70 ± 0.32	1.73 ± 0.31	1.66 ± 0.3
RER	0.99 ± 0.10	0.97 ± 0.10	0.98 ± 0.10	0.95 ± 0.10
*V*_E_ STPD (L · min^-1^)	50.3 ± 11.3	46.0 ± 13.1	47.3 ± 8.8	42.1 ± 8.4
*V*_E_ BTPS (L · min^-1^)	59.6 ± 9.7	56.0 ± 16.0	57.9 ± 11.0	51.4 ± 10.0
Thermoregulatory				
*T*_core_ (**°**C)	37.9 ± 0.2	37.7 ± 0.5	37.9 ± 0.2	37.7 ± 0.3
*T*_skin_ (**°**C)	32.1 ± 1.5	32.1 ± 1.1	32.1 ± 1.5	32.5 ± 1.8
*T*_body_ (**°**C)	36.7 ± 0.3	36.5 ± 0.4	36.8 ± 0.3	36.7 ± 0.5
PSI (AU)	5.1 ± 0.8	4.6 ± 1.4	5.0 ± 0.9	4.6 ± 0.9
Perceptual				
RPE (AU)	15 ± 3	15 ± 2	14 ± 2	14 ± 1
TS (AU)	6 ± 1	5 ± 1	5 ± 1	5 ± 1

### Thermoregulatory responses to the HST

Resting core, skin and body temperatures were unaffected by the previous days exposure (*p* > 0.05). During exercise, core temperature had a tendency to be lower in the HST_HEAT_ and HST_COM_ compared to HST_NORM_ and HST_HYP_ (*p* < 0.05; Figure 6), with medium negative effect sizes in HST_HEAT_ (*d* = -0.63) and HST_COM_ (*d* = -0.69) and a small positive effect size in HST_HYP_ (*d* = 0.26). Mean skin and body temperatures were not different during the exercise period or upon termination of exercise between trials (*p* > 0.05). Plasma volume was significantly increased from day 1 to day 2 in all trials (*p* = 0.004), though no differences were found between experimental conditions (*p* = 0.234). Post exercise changes in plasma volume did not vary between the experimental conditions (*p =* 0.430) (Table 4).

### Physiological strain index

Physiological strain had a tendency to be lower throughout HST_HEAT_ and HST_COM_ compared with HST_NORM_ and HST_HYP_ (*p* = 0.07; Figure 6). When compared to the HST_NORM_, PSI was ≈ 15% lower throughout HST_HEAT_ and ≈ 11% lower throughout HST_COM_, with PSI upon the end of exercise being 10% and 11% lower in these trials, respectively, compared to HST_NORM_. HST_HYP_ had a nominal effect on PSI 24 h later (Figure 6, bottom right panel). PSI during the HST_HEAT_ and HST_COM_ trials was ≈ 10% lower than that during the HST_HYP_ trial. This observation was not statistically significant (*p* = 0.116), though a medium effect size was observed in HST_HEAT_ (*d* = -0.56) and HST_COM_ (*d* = -0.71) but not in HST_HYP_ (*d* = 0.09).

### Ratings of perceived exertion and thermal sensation

Ratings of perceived exertion were not affected by the preceding environmental stressor (*p* = 0.41). Thermal sensation was found to be higher at rest during HST_HEAT_ (*p* = 0.01) and HST_HYP_ (*p* = 0.05), but not different in HST_COM_ (*p* = 0.191) compared to HST_NORM_. At the end of exercise, TS was lower in all experimental conditions compared to HST_NORM_ (*p* < 0.05) (Table 4).

### Monocyte HSP72 responses to HST

mHSP72 had returned to near-baseline values in HST_NORM_ (97% ± 9%) but were elevated from baseline in HST_HEAT_ (130% ± 19%), HST_HYP_ (118% ± 17%) and HST_COM_ (131% ± 19%) (*p* < 0.01; Figure 5). mHSP72 was increased from pre-HST to post-HST in HST_NORM_ (118% ± 12%; *p* < 0.05). This did not occur in any other experimental condition (*p* > 0.05). Large effect sizes were observed for percentage change in post-HST mHSP72 for HST_HEAT_ (*d* = -1.54), HST_HYP_ (*d* = 1.42) and HST_COM_ (*d* = 1.65) when compared with post exercise data in HST_NORM_.

### Plasma pro/anti-inflammatory cytokine responses to the HST

TNF-α remained unchanged 24 h after the initial environmental exposure in all conditions (*p >* 0.05; Figure 5). Plasma IL-10 had returned to near-resting values prior to each of the HST (*p* > 0.05; Figure 5). Post-HST, IL-10 was increased in relation to day 1 baseline values in each condition except HST_COM_ (*p* > 0.05; Figure 5). In relation to the pre-HST sample, IL-10 was elevated post exercise in HST_HYP_ (*p* < 0.05). IL-6 remained elevated 24 h later in all trials compared with initial baseline values (*p* < 0.01; Figure 5), with pre-HST_HEAT_, pre-HST_HYP_ and pre-HST_COM_ values all higher than pre-HST_NORM_ (*p* < 0.01). Pre-HST_HEAT_ and pre-HST_COM_ were higher than pre-HST_HYP_ (*p* < 0.01). Post-HST, IL-6 was increased in all trials (*p* < 0.01). The greatest post exercise increase occurred in HST_HEAT_, whereby IL-6 concentrations were higher than all other post-HST values (*p <* 0.01). In comparison with post exercise values following NORM, HEAT, HYP and COM, IL-6 was higher in HST_NORM_ (*p* < 0.01), reduced in HST_HEAT_ and HST_COM_ (*p <* 0.01) and not different post-HYP and post-HST_HYP_ (*p* > 0.05).

## Discussion

The major findings of this study were that at the levels used to expose participants within this investigation, HEAT induced a greater magnitude of physiological and cellular strain than HYP. The combination of HEAT and HYP induced greater physiological strain than HEAT or HYP alone, supporting the first experimental hypothesis, although post exercise mHSP72 expression was similar between HEAT and COM. A prior acute exposure to HEAT or COM increased basal mHSP72, reduced exercising HR during fixed work hypoxic exercise 24 h later and attenuated the post exercise mHSP72 expression, supporting the second experimental hypothesis. A prior acute exercise bout in hypoxia did not affect hypoxic tolerance 24 h later. On balance, the results suggest that the perturbations to homeostasis induced during an acute heat exposure (40°C) are greater than those resulting from hypoxia (3,000 m). Furthermore, the increased level of systemic strain provided by HEAT had a greater impact on subsequent fixed work exercise in hypoxia, whether hypoxia was a feature of the initial stressor or not.

### Increased physiological strain enhances the preconditioning response

It is well documented in animal models that a prior preconditioning exposure to a stressor, such as heat or ischemia, can improve tolerance and/or survival when later exposed to a different stressor [5]. It has been suggested it is the level of strain, and not solely a stress-specific response, which drives adaptive processes [16]. It is this generalized response to disruptions in homeostasis that may facilitate any preconditioning or cross-acclimation response. It is likely that for a cross-acclimation effect to be present, the variant stressors must share some common acute and adaptive responses [5]. For example, the redistribution of blood flow to the skin during a period of heat stress renders some tissues ischemic. This localized ischemia may also act as a stimulus for induction of HSP72 and also prime the system for later ischemic/hypoxic insult. Of note is the observation that splanchnic tissues undergo ischemia during body heating [27] and that this tissue has been strongly linked with the release of HSP72 into the circulation [28]. It is possible that some of these localized ischemic responses to whole-body heating activate similar cellular and systemic responses which are seen during whole-body hypoxia, and this may play a role in preconditioning and cross-acclimation between heat and hypoxic stressors.

An interesting observation in the present study was that HEAT (40°C) and HYP (F_I_O_2_ ≈ 0.14) produced a similar level of cardiovascular strain during the initial 40 min of exercise (140 ± 8 beats · min^-1^ in HEAT compared to 138 ± 7 beats · min^-1^ in HYP; Figure 3), and each induced post exercise upregulation of mHSP72 and IL-6 (Figure 5), representing both physiological and cellular common responses. However, the magnitude of the cellular stress response was greater in both HEAT and COM and could be due to the greater physiological strain experienced in these conditions (Figure 5). After the initial 40 min of exercise in HEAT, *T*_core_ maintained its rate of rise during HEAT (0.03°C · min^-1^), whereas it plateaued in both NORM and HYP trials from 20 min onwards (Figure 4). The significantly higher heart rate (approximately 12 beats · min^-1^) upon termination of exercise in HEAT compared to HYP was probably due to a reduced ventricular filling time and end-diastolic function (EDV), mediated by central (ANS) or peripheral factors, such as the direct effect of heat on the SA node, increasing the rate of cardiac contraction [29]. As hypothesized, exercise in COM further augmented HR during rest and submaximal exercise (Table 1, Figure 3). During the initial 40 min of exercise in COM, HR was ~10 beats · min^-1^ higher compared to HEAT and HYP (Figure 3), and as a result, PSI was increased throughout exercise in COM compared to both HEAT and HYP (Figure 4). Although the COM exercise condition was, on average, ~6 min shorter than HEAT, final *T*_core_ and PSI were similar (Figure 4), indicating a similar magnitude of overall physiological strain was incurred in these conditions. It is possible to infer that heat *per se* induces the greatest degree of overall cellular strain per unit time due to the increased post exercise mHSP72 and IL-6 seen in both HEAT and COM compared to HYP (Figure 5). The HSR, and expression of HSF-1, was activated in all environmental conditions as evidenced by post exercise mHSP72 expression and the HSF-1-mediated IL-10 increases (Figure 5).

Pre-HST mHSP72 values for HEAT, HYP and COM were similar to, or greater than, those observed post-HST_NORM_ (122% ± 13%). Accordingly, a blunted post exercise HSR was subsequently seen post-HST_HEAT_, post-HST_HYP_ and post-HST_COM_. Previous research has shown that the HSR in monocytes is directly proportional to the amount of HSP72 present in the cell [29]. Conceptually, the monocyte would not require further *de novo* synthesis of mHSP72 as the elevated basal concentrations would allow the cell to cope with HST-induced alterations in cellular homeostasis. IL-10 was, in comparison to the other conditions, unaffected by HST_COM_. The HSR and activation of HSF-1 are implicated in anti-inflammatory responses to stressors [15]; thus, the increased cellular tolerance conveyed as a result of COM may have affected cytokine signal transduction and gene expression via an inhibition of NF-κB, thus preventing expression of the pro-inflammatory mediators such as IL-6 [14]. These results indicate that heat *per se* may induce HSR/HSF-1-mediated anti-inflammatory effects during later hypoxic exercise. Further study should investigate the relationship between HSF-1, HSP72 and both pro- and anti-inflammatory cytokines.

Physiologically, a prior exposure to either HEAT or COM led to modest reductions in exercising HR and *T*_core_ and therefore PSI during their respective HST (Figure 6). In contrast, a prior exposure to hypoxia in the preceding 24 h appeared to have no effect on reducing exercising HR as HR values observed during HST_HYP_ were similar to those in HST_NORM_ (Figure 6). Physiological strain during the HST was also lower following a prior exposure to HEAT and COM in participants 9 and 10, respectively, whereas PSI during exposure to HYP was only reduced in comparison to NORM in 6 out of the 12 participants. This indicates that a prior exposure to a heat stressor improves tolerance to submaximal exercise in hypoxia. Mechanistically, it is possible that an increased plasma volume effect following each heated trial leads to the reduction in HR. No significant statistical change in plasma volume was observed between the four trials; however, each trial resulted in a slight increase in PV 24 h later. It is possible that the duration of exercise impacted on the degree of plasma volume expansion experienced, although in this instance PV expansion was not related to exercise time. Despite this, it does seem the most plausible explanation for the reduction in exercising HR in HST_HEAT_ and HST_COM_. On average, participants had expanded plasma volumes 24 h after HEAT and COM, but not all participants displayed this characteristic. The role physiological strain *per se* has on adaption and subsequent hypoxic tolerance could be further investigated by utilizing a level of hypoxia that induces a greater level of physiological strain than both the levels of HYP and HEAT applied within the present study.

### Exercise in heat offers a more efficient acute training stimulus than hypoxia

The popularity of normobaric altitude training amongst athletes has grown in recent years, despite remaining questions regarding efficacy in improving sea-level performance and performance in hypobaric conditions [30,31]. The results from the current investigation indicate that during an acute fixed work exercise bout, heat presents the greater physiological and cellular training stimulus compared to normobaric hypoxia at the levels studied.

For example, the acute inflammatory response has been shown to play an important role in the response and adaptation to training [32], with IL-6 shown to mediate the metabolic changes during exercise [33]. These results indicate that training at the same absolute workload under conditions of heat stress provides a more potent training stimulus than when performing the same work bout at ~3,000 m asl. It is also inferred that heat induced a greater level of physiological strain at a lower relative workload than acute hypoxia.

As maximal oxygen consumption decreases with increasing altitude [34,35] and increasing ambient temperatures [36,37], absolute workloads under these conditions will be relatively more intense than when performed at sea level. The degree of hypoxia studied in this present investigation has been shown to reduce maximal aerobic capacity to a greater extent than exposure to 40°C heat when compared to values obtained during sea level (HYP = 35% ± 22%, HEAT = 13% ± 11%) [37]. As mean exercise intensity did not vary during the HEAT, HYP and COM trials, it suggests that for the lower relative workload, heat is the greater inducer of IL-6 and mHSP72 and thus represents a greater level of systemic strain than the level of hypoxia studied. These results are aligned with that of Lundby and Steensberg [38] who reported that cycling exercise performed at the same absolute work intensity (50% of normoxic VO_2_ max) at an altitude of 4,100 m elicited a threefold increase in IL-6 compared to that seen at sea level, providing further evidence that exercise intensity augments the IL-6 response [38,39]. Similarly, prolonged cycling for 90 min in the heat at 70% VO_2_ max induced a fourfold increase in IL-6 compared to a normothermic control condition [40]. Heat may offer the greatest practical benefit as an adjunct to training as it elicits a greater physiological and cellular response at a lower, environment-specific workload and for the same level of perceived exertion as experienced in hypoxia (Table 3). Individuals using this approach would also have the option of working at higher work intensities than are possible under hypoxic conditions due to the increased reductions in aerobic capacity experienced in hypoxia. Heat acclimation regimens that elevate plasma volume have been shown to improve physical performance (VO_2_ peak and time trial performance) at sea level in well-trained participants [36,41] and cognitive function during acute hypoxia [18]. Thus, those looking for an adjunct to training may consider the potential benefits of acute and repeated heat training sessions over the more commonly applied altitude model of training.

### Exercise tolerance to fixed work exercise in heat and hypoxia is highly variable

Within- and between-participant exercise capacity was varied between the four environmental stressor conditions (Figure 2). These results confirm data that suggest that aerobic capacity, to some extent, affects exercise tolerance to both heat [42,43] and hypoxia, with those individuals more adapted to endurance exercise better able to regulate their responses to these environmental stressors. These differences become more apparent when participants were separated into trained (>50 mL · kg · min^-1^, 55.8 ± 5.5 mL · kg · min^-1^; *n* = 6) and untrained (<40 mL · kg · min^-1^, 38 ± 2.4 mL · kg · min^-1^; *n* = 6) groups. It is well established that endurance-trained athletes behave physiologically as though already adapted to heat stress [44] via an increased heat loss capacity and decreased rectal temperature [45]. This is illustrated by the slower adaptation to heat seen in those with lower levels of aerobic fitness, compared to their trained counterparts [24]. Heat acclimation has been shown to increase sweat rate and decrease rectal temperature without effecting performance in a trained group of similar aerobic fitness seen in the current investigation (>55 mL · kg · min^-1^), whereas in the untrained group, sweat rate was increased with no changes seen in rectal temperature or exercise performance. The authors concluded that aerobic fitness resulted in significant improvements in exercise heat tolerance, regardless of acclimation status [43]. Thus, the variation in performance seen in this present study may be, in part, related to the training status of participants.

Motivation may have played a factor in the termination of trials, as early termination was not always coincident with a maximal RPE of 20. RPE increased linearly with time in all conditions; however, RPE following the initial 10 min of exercise was higher in the three environmental stress conditions compared to NORM. From a perceptual perspective, one could speculate that heat is a more habitual stressor than hypoxia; thus, natural tolerance and understanding of the physical sensations involved when working under an imposed heat load would be greater than that experienced in hypoxia. None of the participants used in this study had ever been to an altitude of >2,500 m, whereas all had at some stage experienced high ambient temperatures as part of a seasonal variation in climate. Thus, the novel sensations experienced during the hypoxic sessions may have, in part, contributed to cessation of these trials. The increases in skin temperature during the hyperthermic trials and the reductions in arterial oxygen saturation during the hypoxic conditions may have increased the set point for the rate of RPE increase and partially explain some of the differences in exercise capacity observed [46].

### Experimental considerations

The exercise duration during the preconditioning exercise bout on day 1 of each trial may have impacted upon any reductions in HR and *T*_core_. Therefore, future studies employing a similar model are advised to control for exercise duration during the initial bout, ensuring all participants are exposed to the preconditioning stressor for the same length of time. Utilizing fitter participants in future studies may allow for an equal preconditioning dose to be administered across each environmental condition. Alternatively, reducing the exercise intensity may also allow for a consistent exercise dose. This would enable more robust conclusions to be made about the effects of a prior heating exposure on hypoxic tolerance. However, this approach would have compromised the performance capacity aspect during the first stage of this study.

It was important that baseline mHSP72 on the first day of each 2-day trial period did not vary between conditions, as the rate of appearance of HSP72 post heat stress has been shown to be relative to the monocyte basal HSP72 content [47]. The 7-day washout from the end of a HST to the beginning of the next trial allowed resting mHSP72 to return to baseline values. It was not possible to examine the time course of this response, nor was the gene expression profile of HSP72 assessed as part of the current investigation. Morton et al. [26] reported that intramuscular HSP72 peaked at 72 h after a non-damaging running protocol, with values still elevated 7 days after the initial exercise bout. It is possible that the recruitment of a larger muscle mass coupled with eccentric muscle activity may prolong this post exercise elevation in HSP family members compared to the cycling exercise used in the present study. Khassaf et al. [48] utilized a one-legged cycling protocol to elevate intramuscular HSP72. They reported a large inter-individual response to the exercise bout and HSP72 values remaining elevated 3–6 days after exercise. It is therefore possible that each prior trial had a residual effect on intramuscular HSP72 levels that were not reflected in the intracellular samples, collected from the systemic circulation, as part of this investigation. Each experimental block was randomized and completed the trials in different orders, thereby minimizing the potential confounding effects described above. However, the time course of the intramuscular HSP72 response, and how this correlates with systemic intracellular HSP72, warrants further investigation.

## Conclusions

Although exploratory in nature, the results from this study reveal that the levels of heat and hypoxia used produce similar degrees of cardiovascular strain for approximately 40 min of exercise at a work rate of 50% VO_2_ peak. It is anticipated the novel findings of this study will provide a starting point for those interested in investigating different combinations of heat and hypoxia and how these impact upon physical performance. As expected, when heat and hypoxia are combined, acute physiological and cellular stress responses are augmented. However, the level of heat used in this present investigation appears to produce a greater physiological stress response 24 h later compared to the level of hypoxia used, with the combination of two stressors not eliciting greater effects than the use of heat alone. The finding that heat stress *per se* appears to elicit a greater adaptive stimulus than the level of hypoxia studied could have several practical implications. For example, periods of heat training could be implemented into an athlete’s training schedule or be used as an efficient and cost-effective means of preparing individuals (such as military personnel) for rapid redeployment from areas of heat to areas of altitude. Future mechanistic research into short-term, whole-body preconditioning between heat and hypoxia should control for both duration of the initial exposure and degree of hyperthermia induced. The effects of a prior preconditioning period of the whole body or localized muscle heating on exercise tolerance and performance are also a suggested area for future research.

## Abbreviations

ANS: Autonomic nervous system; $a‒\bar{v}O_{2}$: Arterial-venous difference; COM: Combination of heat and hypoxia exercise trial; EDV: End diastolic volume; F_I_O_2_: Fraction of inspired oxygen; HEAT: Heat exercise trial; HR: Heart rate; HSP72: Heat shock protein 72; HSF: Heat shock factor; HSR: Heat shock response; HST: Hypoxic stress test; HYP: Hypoxic exercise trial; IL-6: Interleukin-6; IL-10: Interleukin-10; LPS: Lipopolysaccharide; mHSP72: Monocyte heat shock protein 72; NORM: Normoxic and normothermic exercise trial; PV: Plasma volume; SpO_2_: Arterial hemoglobin oxygen saturation; U_osmo_: Urine osmolality; TNF-α: Tumor necrosis factor 1 alpha; USG: Urine specific gravity.

## Competing interests

The authors declare that they have no competing interests.

## Authors’ contributions

BL participated in the study conception, data collection, sample analysis and statistical analysis and drafted the manuscript. EES participated in the data collection and sample analysis and revised the manuscript. RM assisted with the statistical analysis and participated in the manuscript drafting. AH participated in the data collection and optimization of the flow cytometry assay and revised the manuscript. LT participated in the optimization of the flow cytometry assay and revised the manuscript. RJ participated in the conception of the study, manuscript preparation and manuscript revisions. CDT conceived the study and assisted in the data collection, statistical analysis and manuscript drafting and revisions. All authors read and approved the final manuscript.

## References

[B1] González-Alonso J Crandall CG Johnson JM The cardiovascular challenge of exercising in the heat J Physiol 2008 586 1 45 53 17855754 10.1113/jphysiol.2007.142158 PMC2375553

[B2] Naeije R Physiological adaptation of the cardiovascular system to high altitude Prog Cardiovasc Dis 2010 52 6 456 466 10.1016/j.pcad.2010.03.004 20417339

[B3] Mazzeo R Physiological responses to exercise at altitude Sports Med 2008 38 1 1 8 10.2165/00007256-200838010-00001 18081363

[B4] Tipton M A case for combined environmental stressor studies Extreme Physiol Med 2012 1 1 7 10.1186/2046-7648-1-7 PMC3710159 23849435

[B5] Horowitz M Sharma HS Heat acclimation and cross-tolerance against novel stressors: genomic–physiological linkage Progress in Brain Research. Volume 162 2007 ᅟ: Elsevier 373 392 10.1016/S0079-6123(06)62018-9 17645928

[B6] Taylor L Midgley A Chrismas B Madden L Vince R McNaughton L The effect of acute hypoxia on heat shock protein 72 expression and oxidative stress in vivo Eur J Appl Physiol 2010 109 5 849 855 10.1007/s00421-010-1430-x 20229018

[B7] Taylor L Midgley A Chrismas B Hilman A Madden L Vince R McNaughton L Daily hypoxia increases basal monocyte HSP72 expression in healthy human subjects Amino Acids 2010 ᅟ 1 9 20552383 10.1007/s00726-010-0644-x

[B8] Kregel KC Invited review: heat shock proteins: modifying factors in physiological stress responses and acquired thermotolerance J Appl Physiol 2002 92 5 2177 2186 11960972 10.1152/japplphysiol.01267.2001

[B9] Kuennen M Gillum T Dokladny K Bedrick E Schneider S Moseley P Thermotolerance and heat acclimation may share a common mechanism in humans Am J Physiol Regul Integr Comp Physiol 2011 301 2 R524 R533 10.1152/ajpregu.00039.2011 21613575 PMC3154710

[B10] Taylor L Hillman A Midgley A Peart D Chrismas B McNaughton L Hypoxia-mediated prior induction of monocyte-expressed HSP72 and HSP32 provides protection to the disturbances to redox balance associated with human sub-maximal aerobic exercise Amino Acids 2012 43 5 1933 1944 10.1007/s00726-012-1265-3 22441647

[B11] Sharp FR Ran R Lu A Tang Y Strauss KI Glass T Ardizzone T Bernaudin M Hypoxic preconditioning protects against ischemic brain injury NeuroRx 2004 1 26 35 10.1602/neurorx.1.1.26 15717005 PMC534910

[B12] Mizzen LA Welch WJ Characterization of the thermotolerant cell. I. Effects on protein synthesis activity and the regulation of heat-shock protein 70 expression J Cell Biol 1988 106 4 1105 1116 10.1083/jcb.106.4.1105 3360849 PMC2114998

[B13] Landry J Bernier D Chrétien P Nicole LM Tanguay RM Marceau N Synthesis and degradation of heat shock proteins during development and decay of thermotolerance Cancer Res 1982 42 6 2457 2461 7074623

[B14] Amorim F Moseley PL Asea A, Pederson BK Heat shock protein and inflammation Heat Shock Proteins and Whole Body Physiology 2010 ᅟ: Springer 57 83

[B15] Xiao X Zhang H Tang D Shi Y Gene expression regulation of cytokines by heat shock factor 1 (HSF1) and HSP70 during endotoxemia Shock 2006 25 6 63 64

[B16] Cotter J Cotter JD, Lucas JE, Mundel T Novel stress conditioning for health and performance Proceedings of the 15th International Conference for Environmental Ergonomics: 11th - 15th Feb 2013 2013 Queenstown New Zealand: Publisher: International Society for Environmental Ergonomics 149 150

[B17] Linney M Peplar T Sandhu R Lee BJ Thake CD Physiological responses to lower body positive pressure when walking compared to running Proceedings of the British Association of Sport and Exercise Scientists Annual Conference, Preston: September 3rd - 5th 2013, Journal of Sports Sciences 2014, Volume 32, S65 2013 University of Central Lancaster, UK: Routledge

[B18] Heled Y Peled A Yanovich R Shargal E Pilz-Burstein R Epstein Y Moran DS Heat acclimation and performance in hypoxic conditions Aviat Space Environ Med 2012 83 7 649 653 10.3357/ASEM.3241.2012 22779306

[B19] Durnin J Womersley J Body fat assessed from the total body density and its estimation from skinfold thickness: measurements on 481 men and women aged from 16 to 72 years Br J Nutr 1974 32 77 97 10.1079/BJN19740060 4843734

[B20] Lee BJ Emery-Sinclair E Mackenzie RWA James RS Thake CD Confirmation of an absolute sub-lactate threshold workload for use in studies combining hypoxia and heat stress Proceedings of the British Association of Sport and Exercise Scientists Annual Conference, Preston: September 3rd - 5th 2013, Journal of Sports Sciences 2014, Volume 32, S63 2013 University of Central Lancaster, UK: Routledge

[B21] Ramanathan NL A new weighting system for mean surface temperature of the human body J Appl Physiol 1964 19 3 531 533 14173555 10.1152/jappl.1964.19.3.531

[B22] Stringer WW Hansen JE Wasserman K Cardiac output estimated noninvasively from oxygen uptake during exercise J Appl Physiol 1997 82 3 908 912 9074981 10.1152/jappl.1997.82.3.908

[B23] Moran DS Shitzer A Pandolf KB A physiological strain index to evaluate heat stress Am J Physiol Regul Integr Comp Physiol 1998 275 1 R129 R134 10.1152/ajpregu.1998.275.1.R129 9688970

[B24] Dill DB Costill DL Calculation of percentage changes in volumes of blood, plasma, and red cells in dehydration J Appl Physiol 1974 37 2 247 248 4850854 10.1152/jappl.1974.37.2.247

[B25] Sandstrom M Madden L Taylor L Siegler J Lovell R Midgley A McNaughton L Variation in basal heat shock protein 70 is correlated to core temperature in human subjects Amino Acids 2009 37 2 279 284 10.1007/s00726-008-0144-4 18665435

[B26] Morton JP MacLaren DPM Cable NT Bongers T Griffiths RD Campbell IT Evans L Kayani A McArdle A Drust B Time course and differential responses of the major heat shock protein families in human skeletal muscle following acute nondamaging treadmill exercise J Appl Physiol 2006 101 1 176 182 10.1152/japplphysiol.00046.2006 16565353

[B27] Hall DM Baumgardner KR Oberley TD Gisolfi CV Splanchnic tissues undergo hypoxic stress during whole body hyperthermia Am J Physiol Gastrointest Liver Physiol 1999 276 5 G1195 G1203 10.1152/ajpgi.1999.276.5.G1195 10330010

[B28] Febbraio MA Ott P Nielsen HB Steensberg A Keller C Krustrup P Secher NH Pedersen BK Exercise induces hepatosplanchnic release of heat shock protein 72 in humans J Physiol 2002 544 3 957 962 10.1113/jphysiol.2002.025148 12411538 PMC2290618

[B29] Rubin SA Core temperature regulation of heart rate during exercise in humans J Appl Physiol 1987 62 1997 2002 3597272 10.1152/jappl.1987.62.5.1997

[B30] Muza SR Military applications of hypoxic training for high-altitude operations Med Sci Sports Exerc 2007 39 9 1625 1631 10.1249/mss.0b013e3180de49fe 17805096

[B31] Beidleman BA Fulco CS Staab JE Andrew SP Muza SR Cycling performance decrement is greater in hypobaric versus normobaric hypoxia Extreme Physiol Med 2014 3 8 10.1186/2046-7648-3-8 PMC4002198 24778792

[B32] Petersen AMW Pedersen BK The anti-inflammatory effect of exercise J Appl Physiol 2005 98 4 1154 1162 10.1152/japplphysiol.00164.2004 15772055

[B33] Pedersen BK Febbraio MA Muscle as an endocrine organ: focus on muscle-derived interleukin-6 Physiol Rev 2008 88 4 1379 1406 10.1152/physrev.90100.2007 18923185

[B34] Wagner PD Reduced maximal cardiac output at altitude—mechanisms and significance Respir Physiol 2000 120 1 1 11 10.1016/S0034-5687(99)00101-2 10786640

[B35] Calbet JAL Boushel R Rådegran G Søndergaard H Wagner PD Saltin B Determinants of maximal oxygen uptake in severe acute hypoxia Am J Physiol Regul Integr Comp Physiol 2003 284 2 R291 R303 12388461 10.1152/ajpregu.00155.2002

[B36] Lorenzo S Halliwill JR Sawka MN Minson CT Heat acclimation improves exercise performance J Appl Physiol 2010 109 4 1140 1147 10.1152/japplphysiol.00495.2010 20724560 PMC2963322

[B37] Lee BJ Miller A Owen R Thake CD Comparison of VO_2_ peak between individual and combined environmental stressors Proceedings of the British Association of Sport and Exercise Scientists; Preston September 3rd - 5th 2013, Journal of Sports Sciences 2014, Volume 32, S65 2013 University of Central Lancaster, UK: Routledge

[B38] Lundby C Steensberg A Interleukin-6 response to exercise during acute and chronic hypoxia Eur J Appl Physiol 2004 91 1 88 93 10.1007/s00421-003-0935-y 12955521

[B39] Mazzeo RS Donovan D Fleshner M Butterfield GE Zamudio S Wolfel EE Moore LG Interleukin-6 response to exercise and high-altitude exposure: influence of α-adrenergic blockade J Appl Physiol 2001 91 5 2143 2149 11641355 10.1152/jappl.2001.91.5.2143

[B40] Starkie RL Hargreaves M Rolland J Febbraio MA Heat stress, cytokines, and the immune response to exercise Brain Behav Immun 2005 19 5 404 412 10.1016/j.bbi.2005.03.005 16061150

[B41] Lorenzo S Minson CT Babb TG Halliwill JR Lactate threshold predicting time-trial performance: impact of heat and acclimation J Appl Physiol 2011 111 1 221 227 10.1152/japplphysiol.00334.2011 21527667 PMC3137529

[B42] Selkirk GA McLellan TM Influence of aerobic fitness and body fatness on tolerance to uncompensable heat stress J Appl Physiol 2001 91 5 2055 2063 11641344 10.1152/jappl.2001.91.5.2055

[B43] Cheung SS McLellan TM Heat acclimation, aerobic fitness, and hydration effects on tolerance during uncompensable heat stress J Appl Physiol 1998 84 5 1731 1739 9572824 10.1152/jappl.1998.84.5.1731

[B44] Garrett A Goosens N Rehrer N Patterson M Cotter J Induction and decay of short-term heat acclimation Eur J Appl Physiol 2009 107 6 659 670 10.1007/s00421-009-1182-7 19727796

[B45] Armstrong L Pandolf K Pandolf K, Sawka M, Gonzalez R Physical training, cardiorespiratory fitness and exercise-heat tolerance Human Performance Physiology and Environmental Medicine at Terrestrial Extremes 1988 Indianapolis: Benchmark 199 266

[B46] Crewe H Tucker R Noakes T The rate of increase in rating of perceived exertion predicts the duration of exercise to fatigue at a fixed power output in different environmental conditions Eur J Appl Physiol 2008 103 5 569 577 10.1007/s00421-008-0741-7 18461352

[B47] Vince R Oliver K Midgley A McNaughton L Madden L In vitro heat shock of human monocytes results in a proportional increase of inducible Hsp70 expression according to the basal content Amino Acids 2010 38 5 1423 1428 10.1007/s00726-009-0354-4 19779802

[B48] Khassaf M Child RB McArdle A Brodie DA Esanu C Jackson MJ Time course of responses of human skeletal muscle to oxidative stress induced by nondamaging exercise J Appl Physiol 2001 90 3 1031 1035 11181616 10.1152/jappl.2001.90.3.1031

